# Effects of N-acetyl-L-cysteine on lifespan, locomotor activity and stress-resistance of 3 *Drosophila* species with different lifespans

**DOI:** 10.18632/aging.101561

**Published:** 2018-09-20

**Authors:** Mikhail V. Shaposhnikov, Nadezhda V. Zemskaya, Liubov A. Koval, Eugenia V. Schegoleva, Alex Zhavoronkov, Alexey A. Moskalev

**Affiliations:** 1 Engelhardt Institute of Molecular Biology, Russian Academy of Sciences, Moscow 119991, Russia; 2 Institute of Biology of Komi Science Center of Ural Branch of RAS, Syktyvkar 167982, Russia; 3 Insilico Medicine, Inc, JHU, Rockville, MD 21218, USA; 4 Moscow Institute of Physics and Technology, Dolgoprudny 141700, Russia

**Keywords:** *Drosophila*, lifespan, N-acetylcysteine (NAC), hydrogen sulfide, stress-resistance

## Abstract

N-acetyl-L-cysteine (NAC) is a derivative of the sulphur-containing amino acid L-cysteine with potential anti-aging properties. We studied 3 *Drosophila* species with contrast longevity differences (*D. virilis* is longest-lived, *D. kikkawai* is shortest-lived and *D. melanogaster* has moderate lifespan) to test the effects of NAC at 8 different concentrations (from 10 nM to 100 mM) on the lifespan, stress-resistance and locomotor activity. Except the adverse effects of highest (10 mM and 100 mM) concentrations NAC demonstrated sexually opposite and male-biased effects on *Drosophila* lifespan, stress-resistance and locomotor activity and not satisfied the criteria of a geroprotector in terms of the reproducibility of lifespan extending effects in different model organisms. The concentration- and sex-dependent changes in the relative expression levels of the antioxidant genes (*Cat/CG6871* and *Sod1/CG11793*) and genes involved in hydrogen sulfide biosynthesis (*Cbs/CG1753*, *Eip55E/CG5345* and *Nfs1/CG12264*) suggest the involvement of hormetic mechanisms in the geroprotective effects of NAC.

## Introduction

The search for geroprotectors, compounds that decrease the rate of aging and extend lifespan is one of the important quests in biogerontology and preventive medicine [1–3]. To date, over 200 compounds demonstrated geroprotective effects in model organisms such as yeast S*accharomyces cerevisiae*, nematode *Caenorhabditis elegans*, fly *Drosophila melanogaster* and mouse *Mus musculus* [4,5].

N-acetyl-L-cysteine (NAC) is one of the compounds with the experimentally-confirmed life-extending properties in animal models approved for use in humans [4,5]. NAC supplementation significantly increased the mean and maximum lifespan of *C. elegans* and male *D. melanogaster* [6–8]. Supplementation with NAC also increased lifespan in male *M. musculus* [9]. NAC fully-rescued the lifespan of short-lived *C. elegans* *gas-1*(*fc21*) model of mitochondrial respiratory chain complex I disease toward that of wild-type N2 Bristol worms [10]. In mice with the deficiency of the circadian protein BMAL1 NAC ameliorated the symptoms of premature aging and increased the average and maximum lifespan [11]. NAC significantly increased the lifespan and reduced risk of lymphoma in Atm deficient mice, used as an animal model of ataxia telangiectasia [12].

NAC is a strong antioxidant that elevates glutathione (GSH) production as a precursor of sulphur-containing amino acid L-cysteine and stimulator of the cytosolic enzymes involved in GSH regeneration. NAC also has free radical scavenging activity due to its sulfhydryl (-SH) group oxidation [13,14]. NAC can directly and indirectly modulate multiple signaling pathways affecting cell growth and arrest, apoptosis and inflammation [15–17]. In addition, NAC-derived L-cysteine may stimulate hydrogen sulfide (H_2_S) biosynthesis [18]. Recent study of Ezeriņa et al. [19] demonstrated that NAC protects cells by triggering intracellular H_2_S and sulfane sulfur production. The experimental results suggest a potential role for H_2_S in longevity and stress resistance in such different models as yeast, worms, flies and mice [20,21]. H_2_S influences aging-related signaling pathways by sulfhydration of target proteins and affects cellular bioenergetics, autophagy, inflammation, oxidative stress, proliferation and differentiation of stem cell, cell death and cellular metabolism [22–25].

In the present work, we investigated the effects of NAC supplementation at 8 different concentrations (from 10 nM to 100 mM) on the lifespan, stress-resistance and locomotor activity of 3 *Drosophila* species with different lifespans (*D. melanogaster*, *D. virilis* and *D. kikkawai*) to compare the effects of NAC against of different genotypic background and sex.

We also addressed whether NAC affects the expression level of antioxidant genes (*Cat/CG6871* and *Sod1/CG11793*) and genes involved in H_2_S production (*Cbs/CG1753*, *Eip55E/CG5345* and *Nfs1/CG12264*). The aim of this study was to evaluate the reproducibility of the geroprotective effects of NAC on the individuals of different species and sex for assessing the accordance with the criteria for evaluation of geroprotectors [2] and to investigate the possible role of antioxidant genes and genes involved in H_2_S production in the geroprotective effects of NAC.

## RESULTS

### Lifespan

According to the criteria for evaluation of geroprotectors, their ability to extend lifespan needs to be reproduced in different model organisms [2]. The use of several models with different longevity in one experimental study may reduce the risk of generalizing strain- or species-specific effects of potential geroprotectors. It was previously shown that NAC treatment increases the lifespan of worms, flies and mice [6–11]. However the significant geroprotective effect of NAC was observed only in male but not in female mice [9]. In other models the sex differences in the geroprotective effects of NAC has not been studied yet. To facilitate for multi-species analysis, *D. melanogaster*, *D. kikkawai* and *D. virilis* were chosen to evaluate the sex differences in the effects of NAC. In comparison with *D. melanogaster*, which is characterized by a moderate lifespan (median lifespan: male 54 days, female 67 days; maximum lifespan: male 64 days, female 78 days), *D. virilis* has the longest lifespan (median lifespan: male 71 days, female 75 days; maximum lifespan: male 105 days, female 102 days), while *D. kikkawai* has the shortest lifespan (median lifespan: male 25 days, female 39 days; maximum lifespan: male 36 days, female 51 days) (Supplementary Tables S1-S3). The results of lifespan analysis in these 3 *Drosophila* species are close to our previously published data [26].

**Figure 1:**
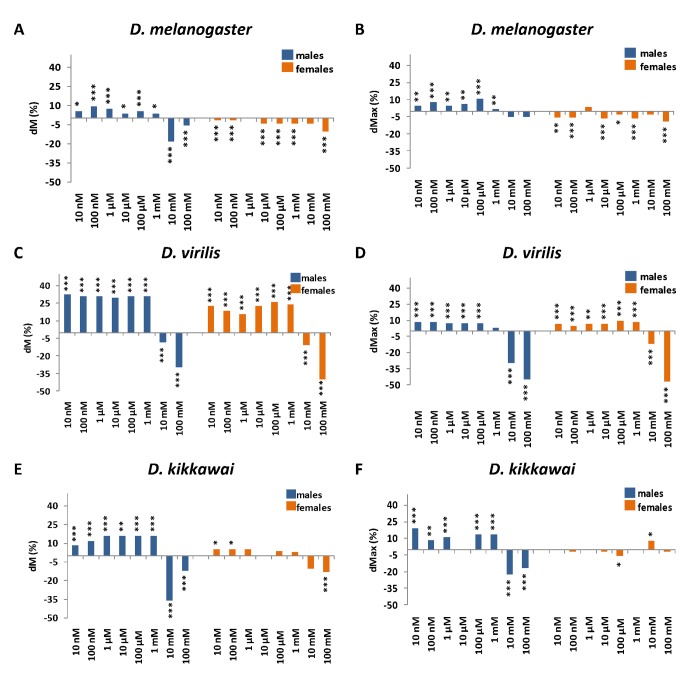
**The effects of NAC supplementation on *D. melanogaster* (A, B), *D. virilis* (C, D), *D*. *kikkawai* (E, F) median (A, C, E) and maximum (B, D, F) lifespan.** dМ, dMax – the differences between median and maximum lifespan of control and experimental flies, respectively. *р<0.05, **р<0.01, ***p <0.001, log-rank test for median lifespan or Wang-Allison test for maximum lifespan.

The effects of NAC supplementation on lifespan parameters are presented in Figure 1, Supplementary Figures S1-S3 and Supplementary Tables S1-S3. The median lifespan of NAC-treated *D. melanogaster* males was increased by 5.6% (p < 0.05), 7.4% (p < 0.001), 5,6% (p < 0.0001) and 3.7% (p < 0.05) at NAC concentrations of 10 nM, 1 μM, 100 μM and 1 mM, respectively. The increase in maximum lifespan of NAC-treated *D. melanogaster* males was 4.7% (p < 0.01), 7.8% (p < 0.0001), 4.7% (p < 0.01), 6.2% (p < 0.01), 10.9% (p < 0.0001) and 1.6% (p < 0.05) for 10 nM, 100 nM, 1 µM, 10 µM, 100 µM and 1 mM NAC, respectively. In contrast to Brack et al. [7] we did not observe any dose-dependent positive effects of NAC on the lifespan. The most significant beneficial effects on median lifespan of *D. melanogaster* males were observed for 100 nM concentration corresponding to 9.3% (p < 0.0001) and on maximum lifespan for 100 μM concentration corresponding to 10.9% (p < 0.0001) (Figure 1A and B, Supplementary Figure S1 and Supplementary Table S1). The higher doses of NAC (10 and 100 mM) were toxic and resulted in adverse effect on median lifespan by 18.5% (p < 0.0001) and by 5.6% (p < 0.001), respectively (Figure 1A and B, Supplementary Table S1). The toxic effects of high NAC doses (20 and 50 mg/ml which approximately corresponds to 120 and 300 mM, respectively) on *D. melanogaster* males correspond with the results previously reported by Brack et al. [7].

In *D. melanogaster* females NAC treatment significantly decreased median lifespan by 1.5% (p < 0.001) for 10 nM and 100 nM NAC and by 4.5% (p < 0.001) for 10 µM, 100 µM and 1 mM NAC (Figure 1A, Supplementary Table S1). NAC supplementation also significantly decreased *the* maximum lifespan of *D. melanogaster* females by 5.1% (p < 0.01) for 10 and 100 nM NAC, by 6.4% (p < 0.001) for 10 µM NAC, by 2.6% (p < 0.05) for 100 µM NAC, by 6.4% (p < 0.0001) for 1 mM NAC and by 9% (p < 0.0001) for 100 mM NAC (Figure 1B, Supplementary Table S1).

In *D. virilis* males NAC treatment increased median lifespan by 32.4% (p < 0.0001) for 10 nM NAC, by 31% (p < 0.0001) for 100 nM, 1 µM, 100 µM and 1 mM NAC and by 29.6% (p < 0.0001) for 10 µM NAC (Figure 1C, Supplementary Figure S2, Supplementary Table S2). NAC supplementation increased the maximum lifespan of *D. virilis* males by 8.6% (p < 0.0001) for 10 nM, by 8.69% (p < 0.0001) for 100 nM, by 7.6% (p < 0.0001) for 1, 10 and 100 µM. However, highest concentrations of NAC decreased median (by 8.5% (p < 0.0001) for 10 mM and by 29.6% (p < 0.0001) for 100 mM) and maximum (by 29.5% (p < 0.0001) for 10 mM and by 44.8% (p < 0.0001) for 100 mM) lifespan of *D. virilis* males (Figure 1D, Supplementary Figure S2, Supplementary Table S2).

In *D. virilis* females NAC treatment increased median lifespan by 22.6% (p < 0.0001) for 10 nM NAC, by 18.7% (p < 0.0001) for 100 nM NAC, by 16% (p < 0.0001) for 1 µM NAC, by 22.7% (p < 0.0001) for 10 µM NAC, by 26% (p < 0.0001) for 100 µM NAC, by 24% (p < 0.0001) for 1 mM NAC. Highest concentrations of NAC decreased median (by 10.7% (p < 0.0001) for 10 mM and by 40% (p < 0.0001) for 100 mM) and maximum (by 11.8% (p < 0.0001) for 10 mM and by 47.1% (p < 0.0001) for 100 mM) lifespan of *D. virilis* females (Figure 1C and D, Supplementary Figure S2, Supplementary Table S2).

In *D. kikkawai* males NAC supplementation increased the median lifespan by 8% (p < 0.0001) for 10 nM NAC, by 12% (p < 0.0001) for 100 nM NAC and by 16% (p < 0.01) for 1 μM, 10 μM, 100 μM and 1 mM. Also, NAC prolonged the maximum lifespan by 19.4% (p < 0.0001) for 10 nM NAC, by 8.3% (p < 0.01) for 100 nM NAC, by 11.1% (p < 0.0001) for 1 μM NAC and by 13.9% (p < 0.0001) for 100 μM and 1 mM NAC (Figure 1E and F, Supplementary Figure S3, Supplementary Table S3). In *D. kikkawai* females NAC supplementation increased the median lifespan 5.1% (p < 0.05) for 10 nM and 100 nM NAC. Treatment of *D. kikkawai* females with 10 mM NAC increased the maximum lifespan by 7.8% (p < 0.05). However, NAC supplementation with the highest concentrations decreased the median (males: by 36% (p < 0.0001) for 10 mM NAC and 12% (p < 0.0001) for 100 mM NAC; females: by 12.8% (p < 0.0001) for 100 mM) and maximum (males: by 22.2% (p < 0.0001) for 10 mM and 16.7% (p < 0.0001) for 100 mM) lifespan in *D. kikkawai* individuals (Figure 1E and F, Supplementary Figure S3, Supplementary Table S3). In addition, treatment of *D. kikkawai* females with 100 μM NAC resulted in decrease in maximum lifespan by 5.9% (p < 0.01).

We concluded that the effects of NAC supplementation on *Drosophila* median and maximum lifespan were species- and sex-specific. In *D. melanogaster* (characterized by a moderate lifespan) the effect of NAC treatment was positive in males (with the exception of the highest concentrations) but negative in females. In *D. kikkawai* (shortest-lived) NAC supplementation prolonged lifespan in males (with the exception of the highest concentrations), but in females geroprotective effect of NAC in low concentrations only was found. In *D. virilis* (longest-lived) NAC increased lifespan of both sexes. The highest concentrations of NAC (10 mM and 100 mM) were toxic and decreased both the median and maximum lifespan of *Drosophila* in most experimental variants. The largest positive effect of NAC supplementation corresponding to about 30% median lifespan extension was observed in *D. virilis* males at NAC concentrations lower than 10 mM. The similar lifespan extending effect in *D. melanogaster* males was obtained by Brack et al. [7] at NAC concentrations of 10 mg/ml (approximately 60 mM). The lifespan extending effect of NAC treatment in other (non-*Drosophila*) model organisms varies from 15-20% with 10 g/l (approximately 60 mM) NAC treatment in *M. musculus* [9] to 30.5% with 5 mM NAC treatment in *C. elegans* [6].

Thus, NAC demonstrated sexually opposite and male-biased effects on *Drosophila* lifespan and not fully satisfies the criteria of a geroprotector in terms of the reproducibility of lifespan extending effects in different model organisms.

### Stress resistance analysis

Long-lived mutants of *S. cerevisiae*, *C. elegans*, *D. melanogaster* and *M. musculus* usually demonstrate high tolerance to the various stress factors, including oxidative stress, starvation, heat shock, cold shock etc [27–30]. Hence, the drug induced increase in stress resistance can serve as an indicator of longevity mechanism activation [2]. In other studies NAC increased resistance to oxidative stress, heat stress, and ultraviolet irradiation in *C. elegans* [6]. In this study we demonstrated that in addition to extending the lifespan, supplementation with NAC affected resistance to oxidative stress (20 mM paraquat), starvation and hyperthermia (33°C).

**Figure 2:**
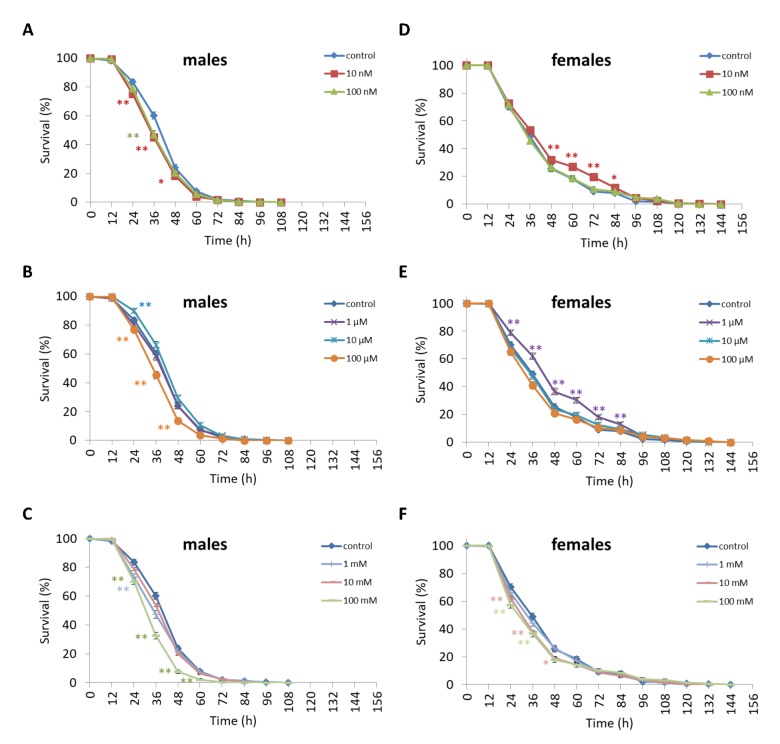
**Influence of NAC treatment on *D. melanogaster* (A, B, C) male and (D, E, F) female survival under oxidative stress (20 mM paraquat).** Results of 3 independent replications are combined. The error bars show standard error of the proportion. *p<0.05, **p<0.01, Fisher’s exact test.

**Figure 3:**
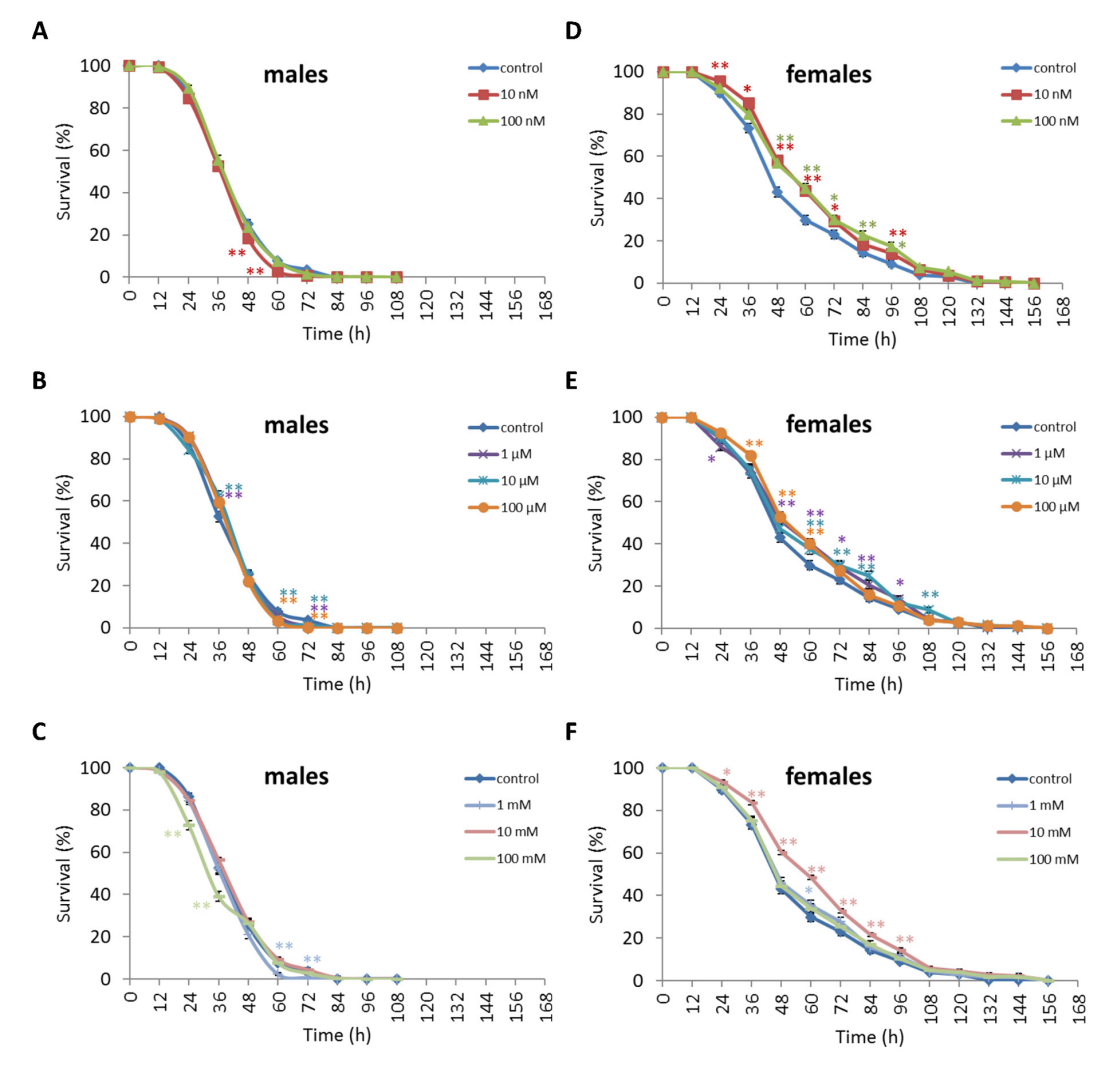
**Influence of NAC treatment on *D. melanogaster* (A, B, C) male and (D, E, F) female survival under starvation.** Results of 3 independent replications are combined. The error bars show standard error of the proportion. *p<0.05, **p<0.01, Fisher’s exact test.

**Figure 4:**
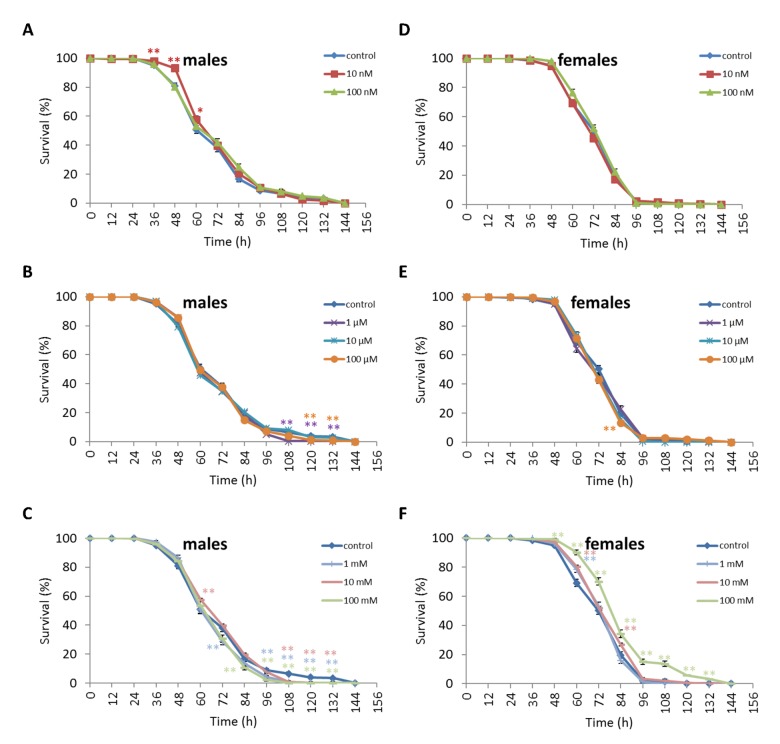
**Influence of NAC treatment on *D. melanogaster* (A, B, C) male and (D, E, F) female survival under hyperthermia (33°C).** Results of 3 independent replications are combined. The error bars show standard error of the proportion. *p<0.05, **p<0.01, Fisher’s exact test.

We observed a decrease in survival rates after 24-48 h of exposure to oxidative stress in *D. melanogaster* males by 3.7-27.5% (p<0.05) when treated with NAC at all concentrations tested except 10 µM, which resulted in increased percentage of surviving male individuals by 5.3-6.6% (p<0.01) after 12 h of exposure to paraquat (Figure 2, Supplementary Table S4). *D. melanogaster* females treated with 10 nM and 1 µM NAC showed increased survival by 4.4-78.8% (p<0.01) after 24-84 h of oxidative stress (Figure 2, Supplementary Table S4). NAC treatment with 10 nM, 1 µM, 10 µM, 100 µM, 1 mM and 100 mM predominantly decreased *D. melanogaster* male survival after 24-72 h starvation by 2.7-13.6% (p<0.01) (Figure 3, Supplementary Table S4). NAC treatment with 10 nM, 100 nM, 10 µM, 100 µM, 1 mM and 10 mM increased *D. melanogaster* female resistance to 24-96 h starvation by 3.6–18.6% (p<0.01) (Figure 3, Supplementary Table S4). The *D. melanogaster* male resistance to 36-60 h hyperthermia was increased after 10 nM NAC by 2.9-12.1% (p<0.01) but decreased to 108-132 h hyperthermia by 2.5-6.4% (p<0.01) after 1 µM, 100 µM and 100 mM NAC. However, in *D. melanogaster* females 100 mM NAC treatment resulted in increased resistance to 60-132 h hyperthermia by 2.9-21% (p<0.01) (Figure 4, Supplementary Table S4).

**Figure 5:**
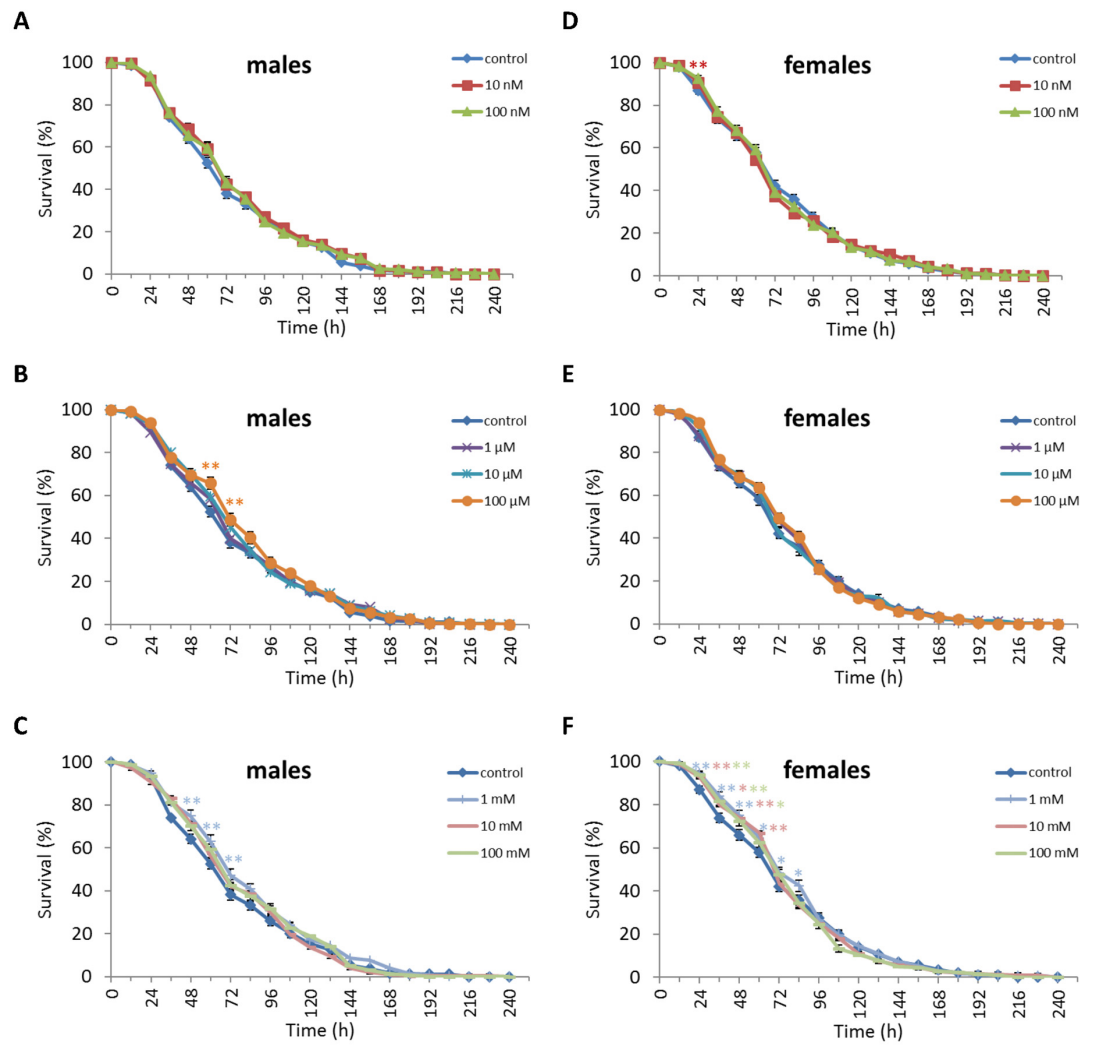
**Influence of NAC treatment on *D. virilis* (A, B, C) male and (D, E, F) female survival under oxidative stress (20 mM paraquat).** Results of 3 independent replications are combined. The error bars show standard error of the proportion. *p<0.05, **p<0.01, Fisher’s exact test.

**Figure 6:**
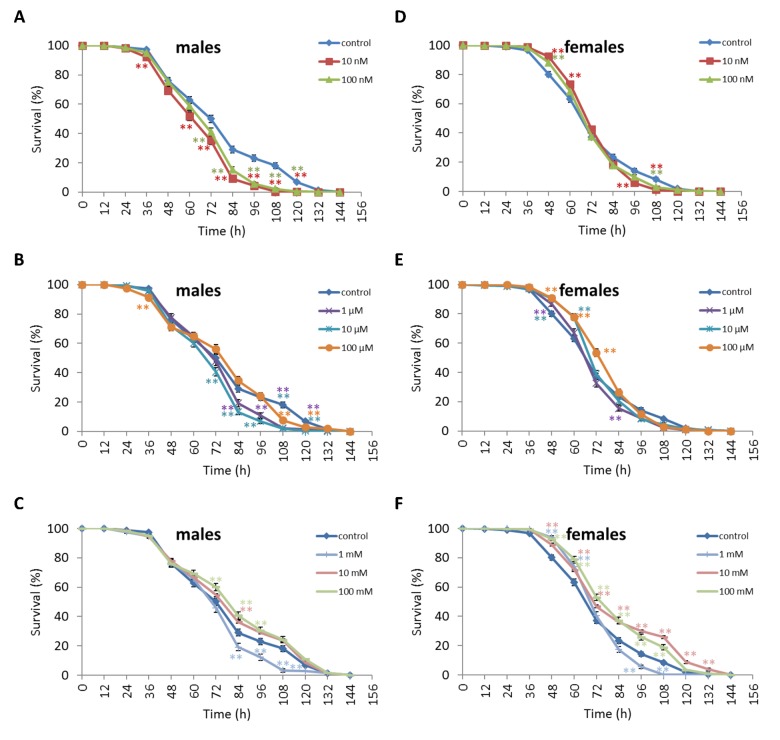
**Influence of NAC treatment on *D. virilis* (A, B, C) male and (D, E, F) female survival under starvation.** Results of 3 independent replications are combined. The error bars show standard error of the proportion. *p<0.05, **p<0.01, Fisher’s exact test.

**Figure 7:**
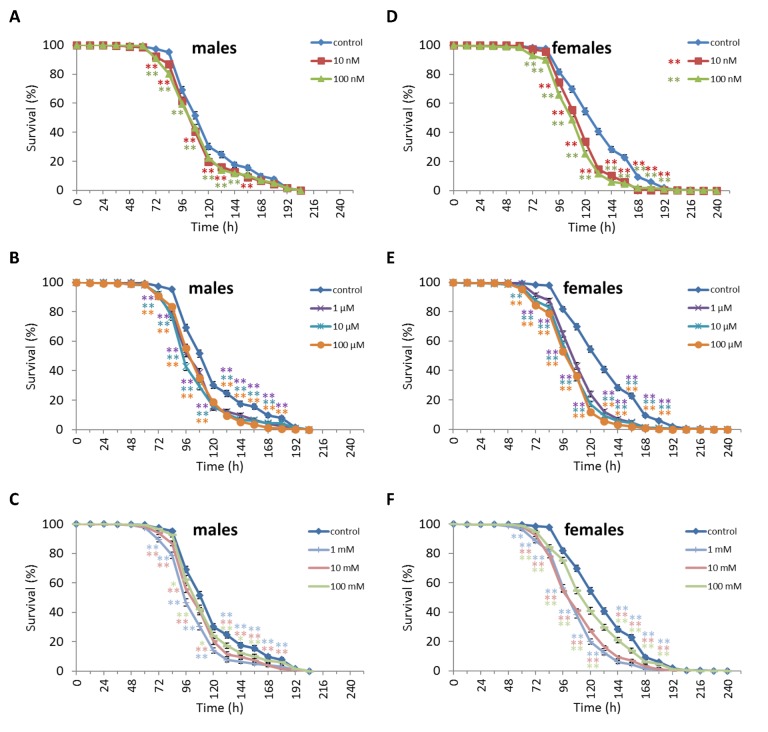
**Influence of NAC treatment on *D. virilis* (A, B, C) male and (D, E, F) female survival under hyperthermia (33°C).** Results of 3 independent replications are combined. The error bars show standard error of the proportion. *p<0.05, **p<0.01, Fisher’s exact test.

Treatment of *D. virilis* males with 100 µM and 1 mM NAC increased survival after 60-72 h of oxidative stress exposure by 8.9-13.3% (p<0.01). Treatment of *D. virilis* females with 1 mM, 10 mM and 100 mM NAC increased resistance to 24-60 h of oxidative stress exposure by 5.8-10.2% (p<0.01) (Figure 5, Supplementary Table S5). *D. virilis* males treated with NAC concentrations lower than 10 mM showed decreased resistance to 36-120 h starvation by 3.9-19.8% (p<0.01), whereas NAC supplementation at 10 mM and 100 mM resulted in increased resistance to 72-96 h starvation by 7.2-11.6% (p<0.01) (Figure 6, Supplementary Table S5). *D. virilis* females treated with any NAC concentration showed increased resistance to exposure to 48-108 h starvation by 3.2-17.5% (Figure 6, Supplementary Table S5). Exposure to any NAC concentration resulted in up to 3.3-26.2% (p<0.01) and 3.6-42.9% (p<0.01) decrease in *D. virilis* male and female survival, respectively, after 72-180 h hyperthermia compared to that of the untreated controls (Figure 7, Supplementary Table S5).

**Figure 8:**
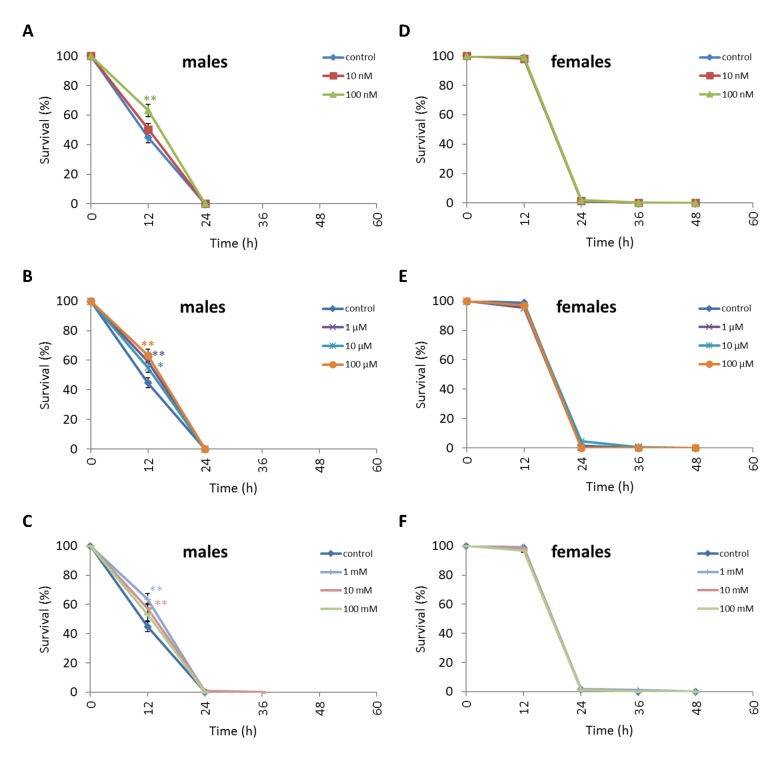
**Influence of NAC treatment on *D. kikkawai* (A, B, C) male and (D, E, F) female survival under oxidative stress (20 mM paraquat).** Results of 3 independent replications are combined. The error bars show standard error of the proportion. *p<0.05, **p<0.01, Fisher’s exact test.

**Figure 9:**
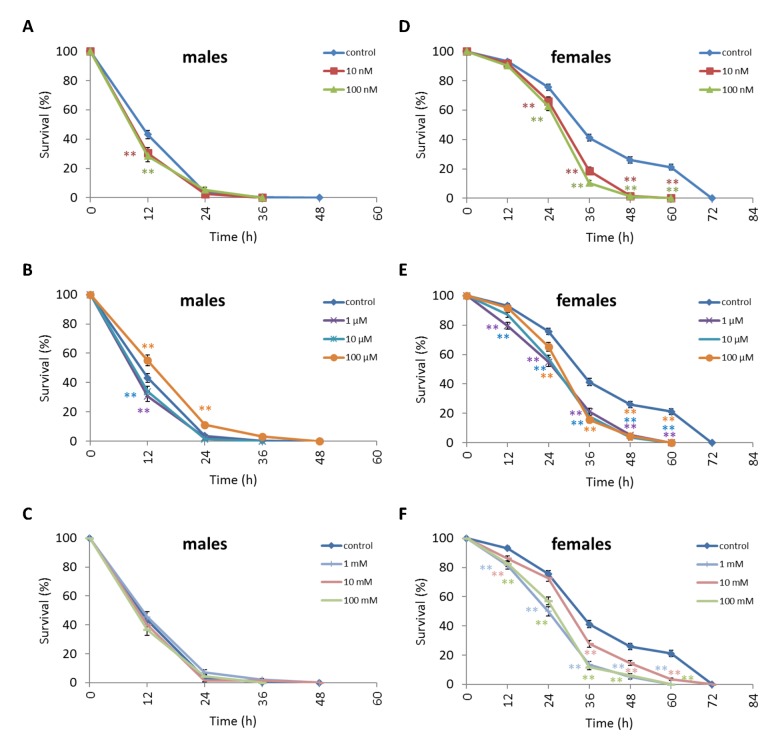
**Influence of NAC treatment on *D. kikkawai* (A, B, C) male and (D, E, F) female survival under starvation.** Results of 3 independent replications are combined. The error bars show standard error of the proportion. *p<0.05, **p<0.01, Fisher’s exact test.

**Figure 10:**
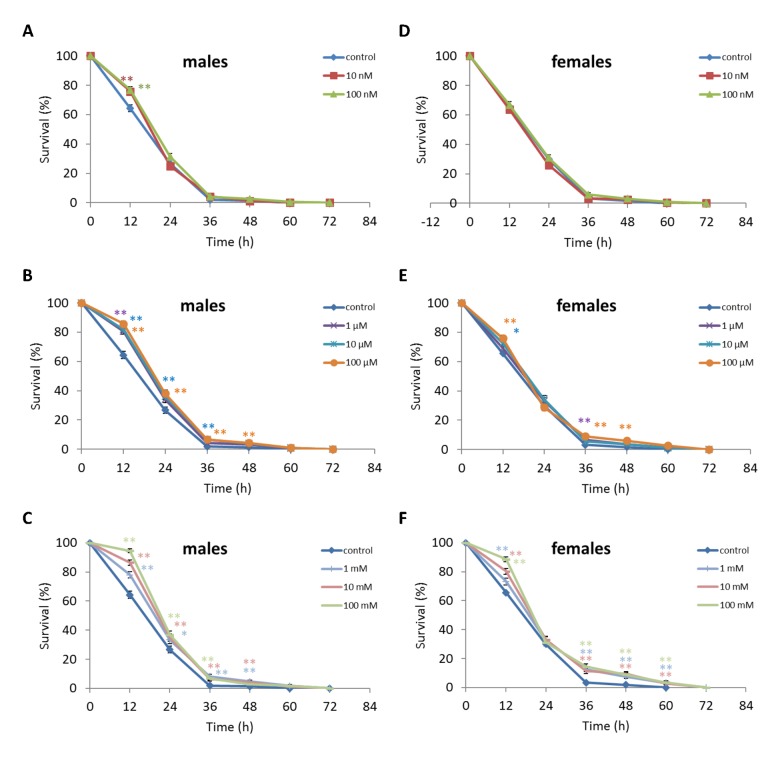
**Influence of NAC treatment on *D. kikkawai* (A, B, C) male and (D, E, F) female survival under hyperthermia (33°C).** Results of 3 independent replications are combined. The error bars show standard error of the proportion. *p<0.05, **p<0.01, Fisher’s exact test.

Treatment of *D. kikkawai* males with any NAC concentration except 10 nM and 100 mM resulted in increased survival after 12 h oxidative stress conditions by 12.6-18.8% (p<0.01) (Figure 8, Supplementary Table S6). The resistance of *D. kikkawai* females to oxidative stress exposure was not affected by NAC supplementation (p>0.05) (Figure 8, Supplementary Table S6). Treatment with 100 µM increased the resistance of *D. kikkawai* male to starvation conditions by 11.9% (p<0.01) after 12 h and by 7.6% (p<0.01) after 24 h (p<0.01) (Figure 9, Supplementary Table S6). Treatment with 10 nM, 100 nM, 1 µM and 10 µM NAC decreased survival under 12 h starvation by 9.3-14.7% (p<0.01). Treatment with all tested concentrations significantly decreased the resistance of *D. kikkawai* females to starvation up to 30.7% (Figure 9, Supplementary Table S6). Treatment with NAC increased resistance to 12-48 h hyperthermia up to 30.3% (p<0.01) and 23.1% (p<0.01) of *D. kikkawai* males and females, respectively (Figure 10, Supplementary Table S6).

In general, the effect of 10 days NAC treatment on the survival under oxidative stress, starvation and hyperthermia was positive in *D. melanogaster* females, but negative in *D. melanogaster* males. NAC treatment had opposite effects on *D. virilis* and *D. kikkawai* male and female resistance to hyperthermia, decreasing survival of *D. virilis*, but increasing survival of *D. kikkawai*.

### Locomotor activity

**Figure 11:**
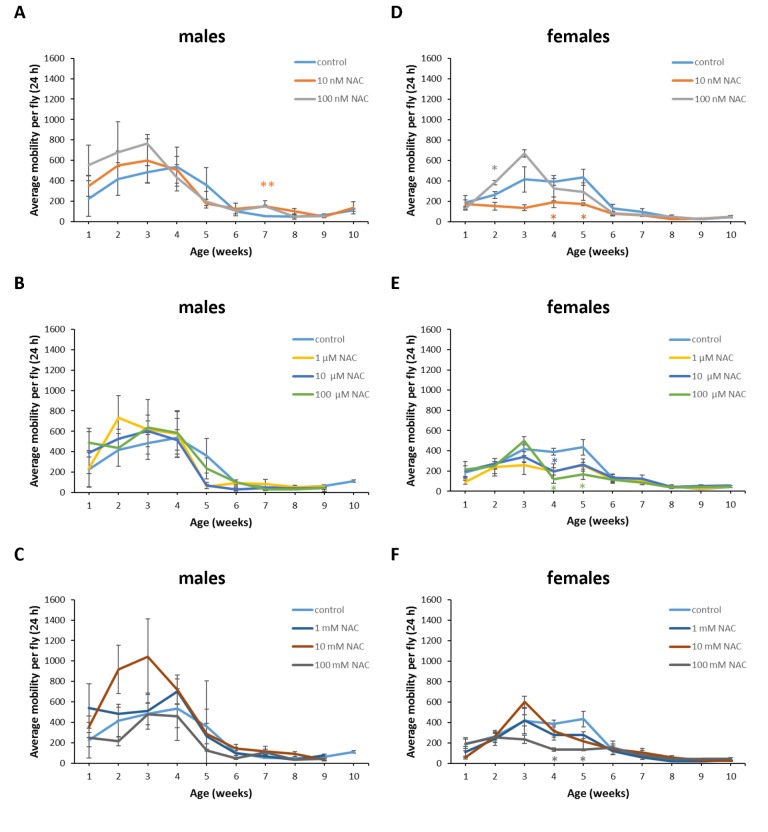
**Influence of NAC supplementation on age-dependent dynamics of total daily locomotor activity of *D*. *melanogaster* males (A, B, C) and females (D, E, F).** The error bars show standard errors. *p<0.05, **p<0.01, t-Student test.

**Figure 12:**
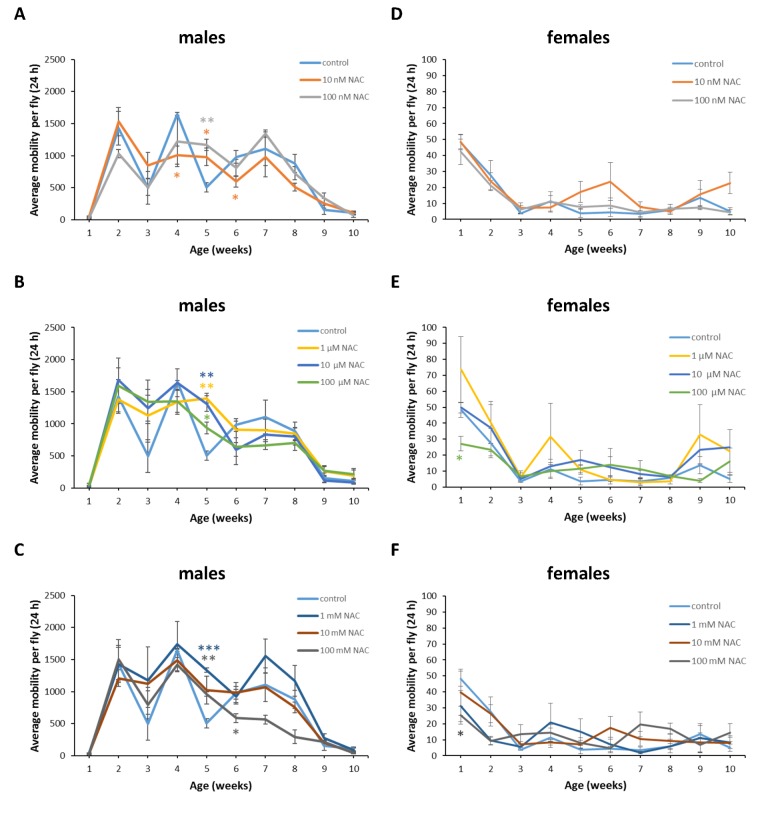
**Influence of NAC supplementation on age-dependent dynamics of total daily locomotor activity of *D. virilis* males (A, B, C) and females (D, E, F).** The error bars show standard errors. *p<0.05, **p<0.01, t-Student test.

**Figure 13:**
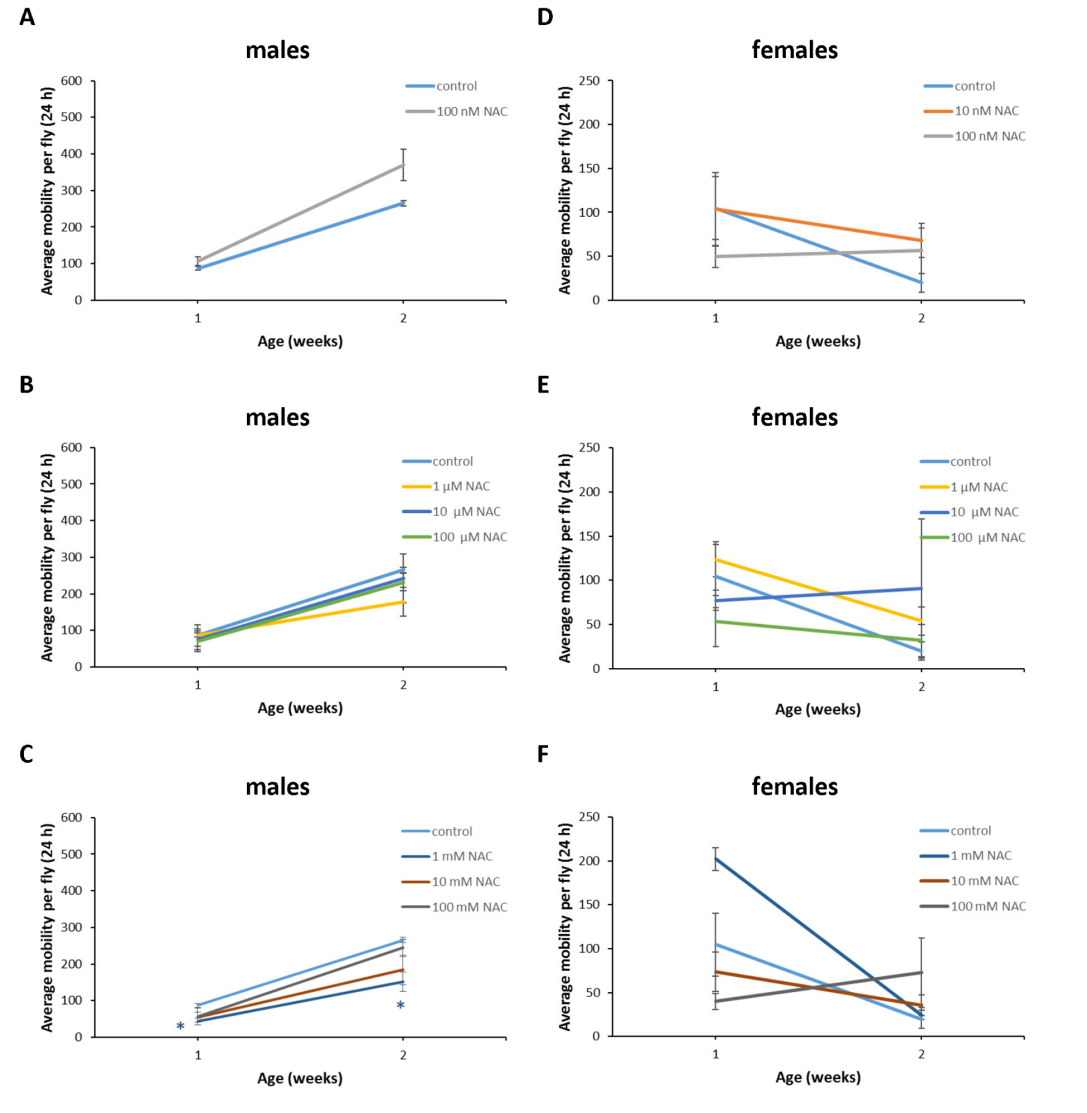
**Influence of NAC supplementation on age-dependent dynamics of total daily locomotor activity of *D. kikkawai* males (A, B, C) and females (D, E, F).** The error bars show standard errors. *p<0.05, **p<0.01, t-Student test.

The locomotor activity is one of the main markers of healthspan [31]. Progressive loss of skeletal muscle function and mass is a common feature of aged humans and model organisms [32]. Previously, we demonstrated that neurospecific overexpression of the pro-longevity gene encoding catalytic subunit (*Gclc*) of glutamate-cysteine ligase (main catalyzer of glutathione synthesis) able to decrease the spontaneous locomotor activity of *D. melanogaster* males and females compared to controls. However, the activity of flies with *Gclc* overexpression did not change during aging [33]. As a GSH precursor, NAC may be of interest as a potential remedy for sarcopenia. Here we used LAM25 Locomotor Activity Monitor (TriKinetics Inc., USA) to measure the influence of NAC treatment with 8 different concentrations on age-dependent changes of total daily locomotor activity in 24 h bins (Figures 11-13) of male and female individuals of 3 *Drosophila* species.

In *D*. *melanogaster* males NAC supplementation with 10 nM resulted in 3 fold (p<0.01) increase in total daily locomotor activity at the age of 7 weeks (Figure 11A). In *D*. *melanogaster* females NAC supplementation with 10 nM, 100 μM and 100 mM decreased total daily locomotor activity at the age of 4 and 5 weeks by 50-70% (p<0.05) approximately (Figure 11D, E, F). At the same time 100 nM NAC-treated *D*. *melanogaster* females demonstrated 46.1% (p<0.05) increase in total daily locomotor activity at the age of 2 weeks (Figure 11D). In *D*. *virilis* males NAC supplementation at all tested concentrations resulted in 1.8-2.8 fold (p<0.05) increase in total daily locomotor activity at the age of 5 weeks (Figure 12A, B, C). NAC supplementation decreased activity at the age of 4 (10 nM) and 6 (10 nM and 100 mM) weeks by about 40% (p<0.05) (Figure 12A, C). In *D*. *virilis* females NAC supplementation with 100 μM and 100 mM decreased total daily locomotor activity at the age of 1 week (Figure 12E, F). In *D*. *kikkawai* males NAC supplementation with 100 mM decreased total daily locomotor activity at the age of 1 and 2 weeks by 50.6% and 43.1%, respectively (Figure 13A, B, C). In *D*. *kikkawai* females NAC supplementation did not affect total daily locomotor activity (Figure 13D, E, F). Thus, NAC supplementation resulted in moderate opposite effects on locomotor activity of *D*. *melanogaster* and *D*. *virilis* males (increased) and females (decreased).

### Expression levels of antioxidant and H_2_S biosynthesis genes

We hypothesized that supplementation with NAC may extend lifespan by promoting free radical detoxification and stimulating the biosynthesis of endogenous H_2_S. Reverse transcription quantitative real-time PCR was performed to evaluate the relative expression levels of *Cat/CG6871* and *Sod1/CG11793* genes encoding a key antioxidant enzymes and genes *Cbs/CG1753*, *Eip55E/CG5345* and *Nfs1/CG12264* involved in H_2_S biosynthesis in *D. melanogaster* males and females after short-term (48 h) NAC treatment*.*

**Figure 14:**
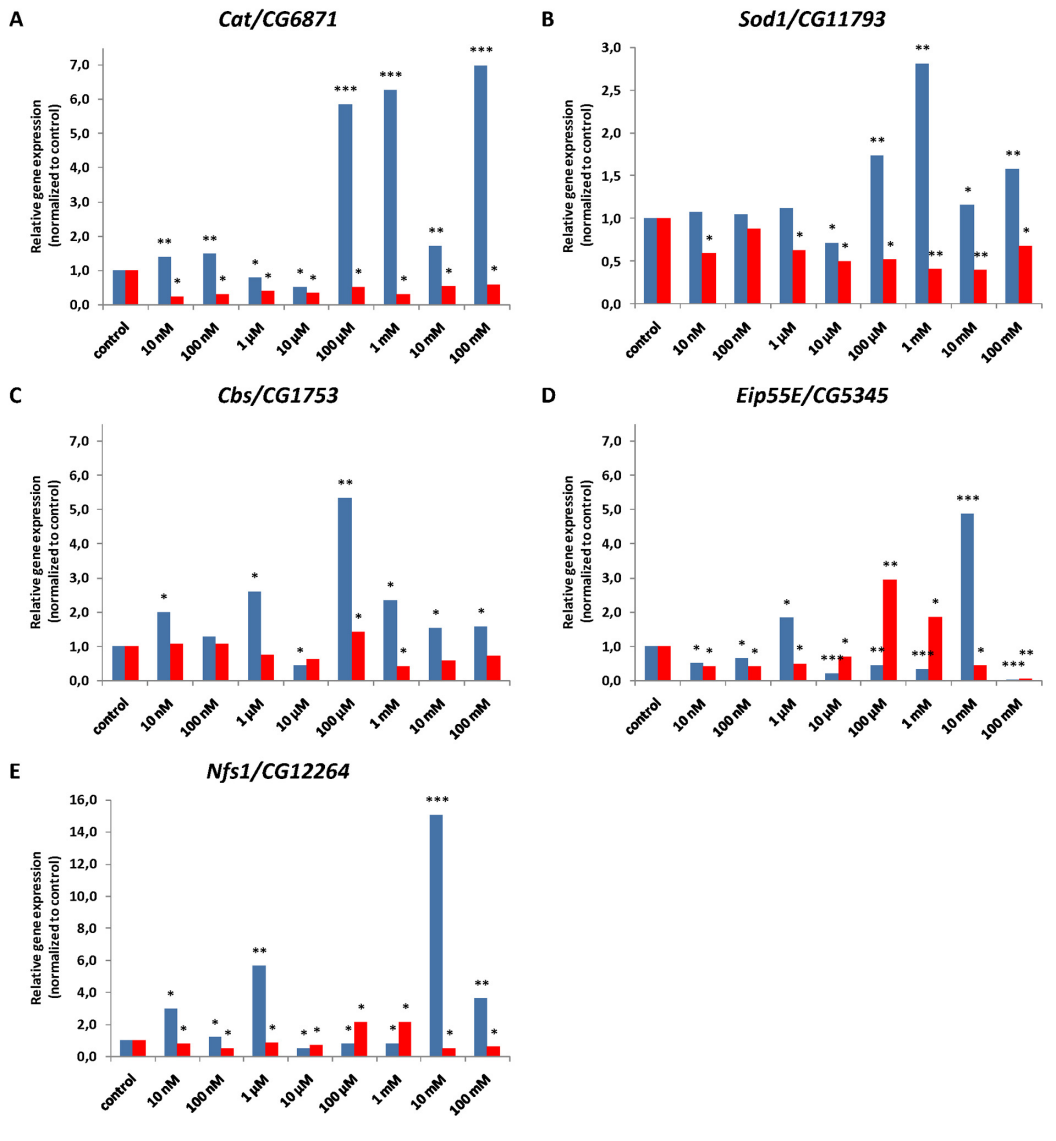
**Relative antioxidant (A, B) and H_2_S biosynthesis (C, D, E) gene expression levels normalized to control in males (blue) and females (red) of *D. melanogaster*.** Results of three independent replications are combined. *p<0.05, **p<0.01, ***p<0.001, t-Student test.

Relative expression level of *Cat/CG6871* gene in males was increased 1.4-6.9 fold (p<0.01, p<0.001) after NAC treatment in most tested concentrations, except 1 μM and 10 μM were it decreased 0.5-0.8 fold (p<0.05) (Figure 14A, Supplementary Table S7). In females all tested NAC concentrations treatment resulted in 0.2-0.6 fold (p<0.05) decrease in expression levels of *Cat/CG6871* gene compared to that of the untreated control (Figure 14A, Supplementary Table S7).

The relative expression level of *Sod1/CG11793* gene in males was unaffected after NAC treatment with 10 nM, 100 nM and 1 μM, decreased 0.7 fold (p<0.05) after 10 μM NAC treatment and increased 1.2-2.8 fold (p<0.01) after 100 μM, 10 mM and 100 mM NAC treatment (p<0.05, p<0.01) (Figure 14B, Supplementary Table S7). After all NAC treatments except 100 nM *Sod1/CG11793* expression in females was 0.4-0.7 fold lower than control value (p<0.05, p<0.01) (Figure 14B, Supplementary Table S7).

NAC is a well-known antioxidant and its stimulating effect on the expression level of antioxidant genes (such as *Cat* and *Sod2*) and GSH level has been demonstrated in various experimental models, including chick omphalocele and hyperoxic mice lung [34,35].

After the first observation of NAC-dependent increase in absolute amounts of total RNA and ribosomal RNA by Brack et al. [7] in *D. melanogaster*, the effects of NAC on the gene expression were examined in different whole organism models. Using *C. elegans* mitochondrial respiratory chain complex I mutant *gas-1*(*fc21*) worms with increased expression of antioxidant genes involving glutathione transferases, alcohol dehydrogenase, and both manganese (mitochondrial, SOD2 homologue) superoxide dismutase and copper-zinc (cytosolic, SOD1 homologue) superoxide dismutase genes, Polyak observed the increase in expression of these genes after 2.5 mM NAC treatment [10]. The green fluorescent protein reporter assay revealed that NAC treatment significantly increased the expression of the *sod-3* genes [6]. Thus our results correspond with the published data.

In animals and in *Drosophila in* particular, H_2_S is produced by two key enzymes, cystathionine β-synthase and cystathionine γ-lyase [20,36]. According to FlyBase (a database for *Drosophila* genetics and molecular biology) 3 genes responsible for H_2_S production in *Drosophila* were identified. Among these genes *Cbs/CG1753* that is the single homolog of *CBS* (encodes cystathionine β-synthase) [20], *Eip55E/CG5345* that codes the cytoplasm-located orthologue of *CSE* (encodes cystathionine γ-lyase) [36] and *Nfs1/CG12264* coding for mitochondria-located enzyme with cystathionine γ-lyase activity [37].

We found that *Cbs/CG1753* gene expression in males treated with 10 μM NAC was 0.4 fold (p<0.05) lower than control level. In other experimental variants *Cbs/CG1753* expression level increased 1.5-5.3 fold (p<0.05, p<0.01) (except unaffected variant of 100 nM NAC) (Figure 14C, Supplementary Table S7). In females 100 μM NAC treatment resulted in 1.4 fold (p<0.05) increase in *Cbs/CG1753* expression relative to control, 1 mM NAC - in 0.4 fold decrease (p<0.05), while no significant effect occurred with other concentrations (Figure 14C, Supplementary Table S7).

The relative expression level of *Eip55E/CG5345* gene in males was 1.8 and 4.9 fold higher after 1 μM and 10 mM NAC treatment, respectively (p<0.05, p<0.001) and 0.03-0.5 fold lower in other variants (p<0.05, p<0.01, p<0.001). The 1 mM and 100 μM NAC treatment resulted in 1.9-2.9 fold (p<0.05, p<0.01) increase in *Eip55E/CG5345* gene expression level respectively in females. In other variants *Eip55E/CG5345* expression was decreased 0.1-0.7 fold (p<0.05, p<0.01) (Figure 14D, Supplementary Table S7).

The relative expression level of *Nfs1/CG12264* gene in males was increased 1.2-15 fold (p<0.05, p<0.01, p<0.001) after NAC treatment with 10 nM, 100 nM, 1 μM, 10 mM and 100 mM. NAC treatment with 10 μM and 100 μM, 1 mM resulted in 0.5-0.8 fold (p<0.05) decrease in *Nfs1/CG12264* expression (Figure 14E, Supplementary Table S7). *Nfs1/CG12264* expression in females was 2.1 fold (p<0.05) increased with 100 μM and 1 mM NAC, but 0.5-0.8 fold (p<0.05) decrease in other variants (Figure 14E, Supplementary Table S7).

In summary, we observed predominantly the opposite effects of NAC supplementation on gene expression in males and females. In most experimental variants, a significant (up to 15 fold) increase in the expression level of the studied genes was observed in males while it was decreased or unaffected in females.

## DISCUSSION

In this study, we analyzed the effects of NAC treatment at 8 different concentrations (from 10 nM to 100 mM) on the lifespan, stress resistance (paraquat, hyperthermia, starvation) and healthspan (locomotor activity) of 3 *Drosophila* species. We selected these species for the substantial difference in their lifespans: *D. melanogaster* is characterized by a moderate lifespan, *D. virilis* is longest-lived and *D. kikkawai* is shortest-lived [26].

In previous study of transcriptomes of 14 *Drosophila* species with different lifespans among the genes with positive correlation to longevity *Gclm* was found [26]. *Gclm* codes for the modulatory subunit of glutamate-cysteine ligase (GCL), the rate-limiting enzyme in *de novo* glutathione biosynthesis [38]. Overexpressing either the catalytic (GCLc) or modulatory (GCLm) subunit of GCL increased glutathione content in fly homogenates and extended mean and maximum lifespans, increased resistance to oxidative, proteotoxic and osmotic stresses, slowed down the age-dependent decline of locomotor activity and circadian rhythmicity without effect on fecundity [33,38]. Therefore, increasing glutathione level with NAC treatment resemble this naturally selected mechanism of stress-resistance and longevity. However, we observed that the effects of NAC supplementation on lifespan, stress resistance and healthspan were species- and sex-specific.

Sex differences in response to life-extending genetic or pharmacological interventions widespread in different model organisms [39,40]. It is typical for both flies and mice that dietary restriction, reduced insulin/IGF1-like signaling, and inhibited TOR signaling increase lifespan preferentially in females, whereas longevity enhancement due to a range of pharmacological treatments favors males [39,40]. The mechanisms of sex-specific and sex-biased effects of anti-aging interventions have not yet been fully elucidated, but it may be influenced by sex-specific gene expression, sex differences in mitochondrial maintenance failure and sex steroids [39,40].

As recently revealed in comprehensive metabolome analyses of systemic lupus erythematosus (SLE) patients, NAC blocked mechanistic target of rapamycin (mTOR) activation [41]. According to Mikhail Blagosklonny hypothesis [42] overactivated mTOR may render young males robust at the cost of accelerated aging. In this case mTOR inhibition by NAC may slow male aging and increase lifespan.

The mTOR activity in SLE patients was stimulated by accumulation of kynurenine (KYN), metabolite of the amino acid L-tryptophan (TRY), but NAC treatment significantly reduced KYN level [41]. Upregulation of KYN formation from TRY was associated with aging and neurodegeneration in animal and human studies [43,44], while pharmacological inhibition of TRY-KYN metabolism prolonged mean and maximum lifespan in *D. melanogaster* females [45] and may have therapeutic relevance in neurodegenerative disorders [43,44]. However, inhibition of TRY-KYN metabolism by NAC has not yet been studied experimentally on the *Drosophila* model.

To date, it is widely accepted that lifespan-extending effect of NAC associated with antioxidant activity [6–9].

We have shown that relative *Cat/CG6871* and *Sod1/CG11793* genes expression levels were predominantly increased in males but decreased in females. Transgene overexpression of *Cat* and *Sod1* gene have been found to result in a 33% lifespan extension, a slower rate of mortality acceleration and a delayed loss in physical performance in *D. melanogaster* [46]. These results are consistent with the observed male-biased effects of NAC on lifespan, and suggest that antioxidant activity of NAC can be mediated by activation of antioxidant genes expression.

It is worth noting that 1 mg/ml (about 6 mM) NAC supplementation increases mitochondrial hydrogen peroxide (H_2_O_2_) levels in various *Drosophila* tissues [47]. Albrecht et al. [47] also observed that longer lifespan in *Drosophila* correlated with accelerated accumulation of cytosolic H_2_O_2_ in the gut. The green fluorescent protein (GFP) reporter assay revealed activation of the expression of stress-responsive genes, *sod-3* and *hsp-16.2*, following NAC treatment of *C. elegans* [6].

These data indicate that NAC treatment can modificate the mechanisms of hormesis (beneficial effects of low doses of toxic substances) [48]. The hormesis involve activation of such stress-responsive mechanisms as heat shock proteins, antioxidant enzymes, DNA repair machinery and immune response [49–52]. Number of known pharmacological agents promote model organisms’ longevity via hormetic mechanisms [4,5]. For example, the lifespan-extending effect of carotenoid fucoxanthin in *D. melanogaster* is associated with upregulation of genes involved in heat shock response (*hsp70Aa*), oxidative stress (*sod1*, *Keap1*, *CncC*/*Nrf2*, *GclC*) and DNA repair (*D-GADD45*, *mei-9*, *spn-B*, *p53*) [53]. The dietary pectins increases lifespan in *D. melanogaster* and activates the expression of genes involved in heat shock response (*hsp70Aa*), DNA repair (*D-GADD45*, *mei-9*, *spn-B*) and apoptosis (*wrinkled*/*hid*) [54].

Recently, the possible role of NAC-derived L-cysteine in the H_2_S biosynthesis was suggested [18]. Ezeriņa et al. [19] demonstrated that NAC triggers intracellular H_2_S and sulfane sulfur production that provide antioxidative and cytoprotective effects. In this study we demonstrated the relationship between the geroprotective effects of NAC supplementation and activation of genes involved in H_2_S biosynthesis. In animals and particular in *Drosophila*, H_2_S is produced by two key enzymes, cystathionine β-synthase (CBS) and cystathionine γ-lyase (CSE) [20,36]. We have shown that NAC predominantly increased relative *Cbs/CG1753* gene expression in *D. melanogaster* males but not significantly affected or decreased its relative expression level in females. The positive effects of NAC-treatment on the relative expression levels of *Eip55E/CG5345* and *Nfs1/CG12264* genes were also more pronounced in males compared to the females. The observed results of H_2_S biosynthesis genes activation are consistent with the male-biased effects of NAC on lifespan. It is noteworthy that intermediate concentrations of NAC (10 μM, 100 μM and 1 mM) decreased expression level of *Nfs1/CG12264* gene in males while lower (10 nM, 100 nM, 1 μM) and higher (10 mM and 100 mM) concentrations increased *Nfs1/CG12264* expression. This U-shaped dose response supports the hormetic mechanisms of the NAC effects [55].

The activity of CBS was up-regulated in flies exposed to dietary restriction (DR), and transgene-mediated increases in gene expression and enzyme activity of CBS was sufficient to increase lifespan in fully fed *Drosophila* [20]. However, RNAi-mediated knockdown of *CBS* or inhibition of the CSE using propargylglycine limited or abrogated lifespan extension by diet [20]. Overexpression of *CSE* in *Drosophila* model of spinocerebellar ataxia type 3 (SCA3) restored protein persulfidation, decreased oxidative stress, dampened the immune response and improved SCA3-associated tissue damage and neurodegeneration [56]. Overexpression of *CBS-1* in nematodes *C. elegans* increased lifespan independent of diet, while knocking down the same gene abrogated DR-mediated lifespan extension [21]. In mice model of homocysteinemia, completely lacking of cystathionine β-synthase as a result of homozygous mutation in the *CBS*, severe growth retardation and significantly reduced lifespan (about 5 weeks) were observed [57]. Mice with a targeted deletion of the *CSE* also demonstrated increased plasma homocysteine level, growth retardation and exhibit a shortened lifespan (about 12 weeks) on a cysteine-limited diet [58].

Thus we observed that the effects of NAC supplementation on lifespan, stress resistance and healthspan were species- and sex-specific. NAC demonstrated sexually opposite and male-biased effects on *Drosophila* lifespan and not fully satisfied the criteria of a geroprotector in terms of the reproducibility of lifespan extending effects in different model organisms. In this regard, we should not exclude the possibility of the sex-specific effects of NAC on the life span of other model organisms and humans. We also shown that NAC activates the transcription level of the antioxidant genes and genes involved in hydrogen sulfide biosynthesis in a concentration- and sex-specific manner. The obtained results suggest the involvement of hormetic mechanisms in the geroprotective effects of NAC.

## MATERIALS AND METHODS

### *Drosophila* strains

In this study we used 3 *Drosophila* species with differences in lifespan: *D. melanogaster*, characterized by a moderate lifespan, *D. virilis* with the longest lifespan and *D. kikkawai* with the shortest lifespan [26]. *D. melanogaster* wild type *Canton-S* line was obtained from Bloomington Stock Center at Indiana University (#64349, Bloomington, USA). *D. virilis* (#15010–1051.87) and *D. kikkawai* (#14028–0561.14) strains originally derived from the *Drosophila* Species Stock Center at Cornell University (La Jolla, USA) were provided by Vadim Gladyshev (Harvard Medical School, USA).

### Treatment with N-acetyl-L-cysteine

All of the 3 fly species were maintained on a sugar-yeast medium with a spread of yeast paste (50 g of inactivated by heating dry yeast per 60 ml of water). Starting from the first day of life, experimental flies were fed by a yeast paste with NAC (Sigma-Aldrich, USA) in concentration of 10 nM and 100 nM, 1 μM, 10 μM and 100 μM, 1 mM, 10 mM and 100 mM. A 100× concentrated stock solutions NAC in H_2_O and chilled fresh yeast paste were used to prepare the food for all of the NAC concentrations except 10 mM and 100 mM. To prepare yeast paste with 10 mM and 100 mM of NAC, it was added directly to the paste without being dissolved in H_2_O. Treatment was continued until all flies died. Control animals were fed with yeast paste without NAC or other additives. Flies were transferred to a fresh medium vials without anesthesia 2-3 times a week.

### Lifespan analysis

Newly eclosed flies were collected within 24 h and sorted by sex using light carbon dioxide (CO_2_) anesthesia (Genesee Scientific, USA). Animals were housed in a constant climate chamber Binder KBF720-ICH (Binder, Germany) at 25°C and at 60% relative humidity under a 12 h : 12 h light/dark cycle. All flies were maintained on standard sugar-yeast medium in a *Drosophila* narrow vials (Genesee Scientific, USA) at a density of 30 males or females per vial, with 5 vials per experimental variant. Dead flies were counted daily. Experiments were performed in 2 replicates. The median and maximum (age of 90% mortality) lifespan and the mortality rate doubling time (MRDT) were calculated.

### Stress resistance analysis

To investigate the effect of NAC feeding on the resistance to oxidative stress, starvation and hyperthermia, the newly eclosed male and female flies were collected and fed a diet with or without the NAC for 10 days. To assay resistance to oxidative stress, flies were exposed to medium composed of 2% agar, 5% sucrose and 20 mM paraquat (Sigma-Aldrich, USA). During starvation the flies were kept on 2% agar medium. Hyperthermia was induced by continuous exposure of the flies to 33°C. Dead flies were counted twice a day until the end of the experiment. The survival rates of control and NAC-fed flies after different times of exposure to stress factors were calculated. In each experimental variant 3-5 vials of 30 flies of each sex were used. All experiments were carried out in 3 replicates.

### Analysis of locomotor activity

The influence of NAC on the age-dependent changes in spontaneous locomotor activity was measured using the LAM25 Locomotor Activity Monitor (TriKinetics Inc., USA) under standard 12 h lights on, 12 h lights off conditions. The data from 10 flies in 3 vials as replicates were collected during 24 h and represented as average total daily locomotor activity. Measurements were carried out every week, from the age of 1 to 2-10 weeks, while the sufficient number of flies remained alive in control and experimental variants.

### qRT-PCR

Freshly emerged *D. melanogaster* imagoes were collected within 24 h and treated with NAC for 48 h. The gene expression analyses were carried out using whole bodies of 20 males or 10 females per variant of experiment. Reverse transcription quantitative real-time PCR (qRT-PCR) were used to measure the expression levels of antioxidant genes: *Catalase* (*Cat/CG6871*), *Superoxide dismutase 1* (*Sod1/CG11793*) and genes involved in H_2_S production: *Cystathionine β-synthase* (*Cbs/CG1753*), *Ecdysone-induced protein 55E* (*Eip55E/CG5345*) and *Nfs1 cysteine desulfurase* (*Nfs1/CG12264*). RNA was isolated by Aurum Total RNA mini kit (Bio-Rad, USA). To determine total RNA concentration was used Quant-iT RNA Assay Kit (Invitrogen, USA). Reverse transcription was performed using the iScript cDNA Synthesis Kit (Bio-Rad, USA). The mix for RT-PCR was prepared by iTaq Universal SYBR Green Supermix (Bio-Rad, USA) with primers listed in Supplementary Table S8. The primer design was performed using QuantPrime online tool [59]. The reaction was carried out on the CFX96 Real-Time PCR Detection System (Bio-Rad, USA) using the following parameters: one cycle of 95 °C for 30 s; 40 cycles of 95 °C for 10 s and 60 °C for 30 s. Expression levels of target genes was calculated relative to the expression of reference gene (*β-Tubulin*) [60] using the CFX Manager software (Bio-Rad, USA). Experiments were made in 3 independent biological replicates, with 3 technical replicates in each.

### Statistical analysis

To compare the statistical differences in median lifespan between control and experimental groups, the log-rank test was used [61]. A Wang-Allison test was used to estimate the differences in the maximum lifespan (age of 90% mortality) [62]. The comparison of survival functions was done using the modified Kolmogorov-Smirnov test [63]. To assess the statistical significance of differences in resistance to stress factors, the Fisher's exact test was used [64]. To compare the statistical significance of locomotor activity relative and gene expression levels between control and experimental flies, t-Student test was used. The statistical analyses of the data were carried out using STATISTICA software, version 6.1 (StatSoft, USA), R, version 2.15.1 (The R Foundation) and OASIS 2: Online Application for Survival Analysis 2 [64].

## Supplementary Material

Supplementary Figures

Supplementary Tables

Supplementary Table S4

Supplementary Table S5

Supplementary Table S6
