# Supplementary material for: Effects of N-acetyl-L-cysteine on lifespan, locomotor activity and stress-resistance of 3 Drosophila species with different lifespans
Source: Aging (Albany NY). 2018 Sep 20;10(9):2428–58. doi: 10.18632/aging.101561 (PMC6188487; doi:10.18632/aging.101561)
Supplement: Supplementary Figures [file aging-10-101561-s001.pdf]

SUPPLEMENTARY MATERIAL

SUPPLEMENTARY FIGURES

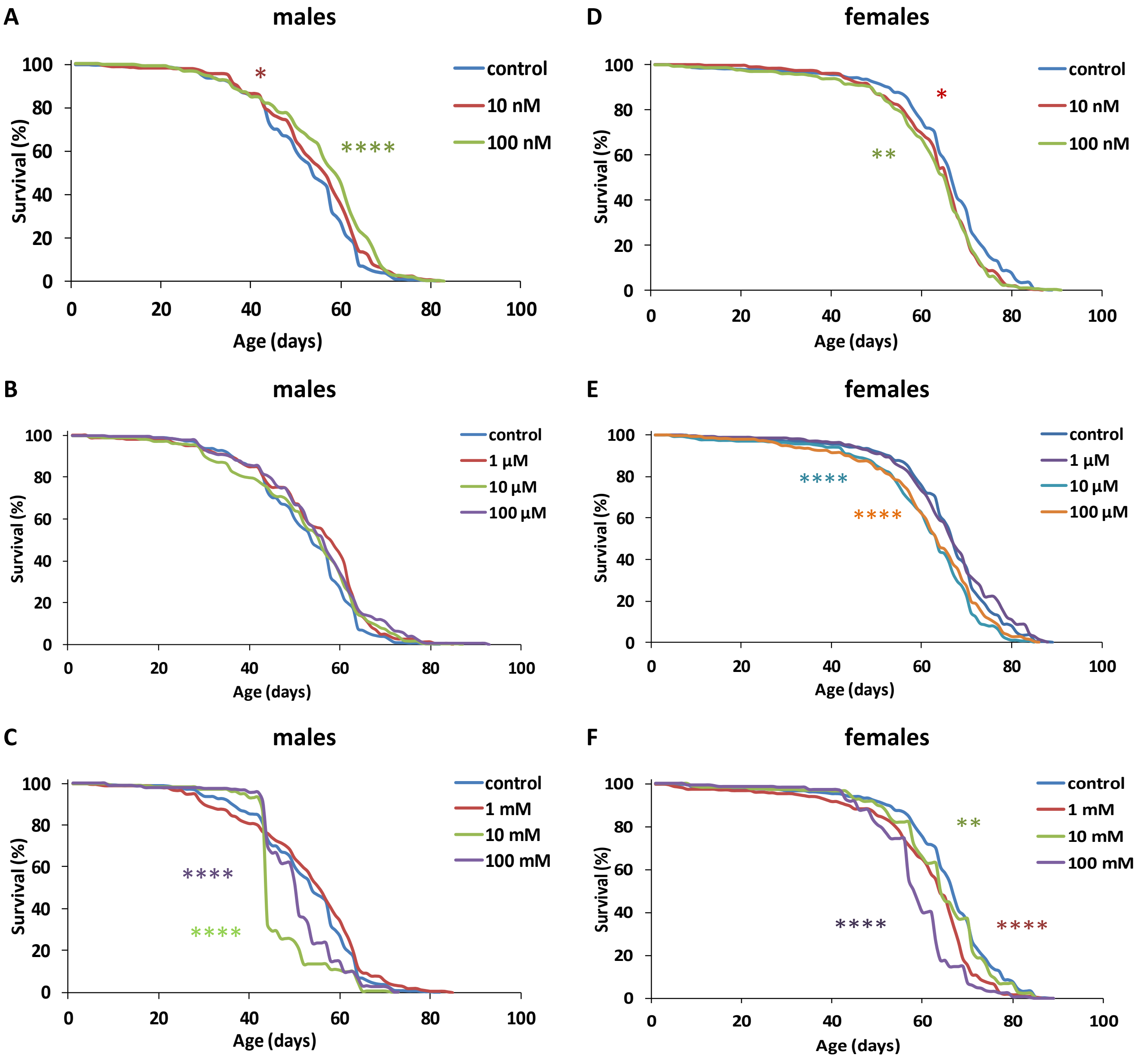

Supplementary Figure S1. Influence of NAC supplementation with nano- (A, D), micro- (B, E) and (C, F) millimolar concentrations on the *D. melanogaster* male (A, B, C) and female (D, E, F) lifespan (combined data of two independent replications). $^{*}p<0.05$, $^{**}p<0.01$, $^{***}p<0.001$, $^{****}p<0.0001$, Kolmogorov–Smirnov test.

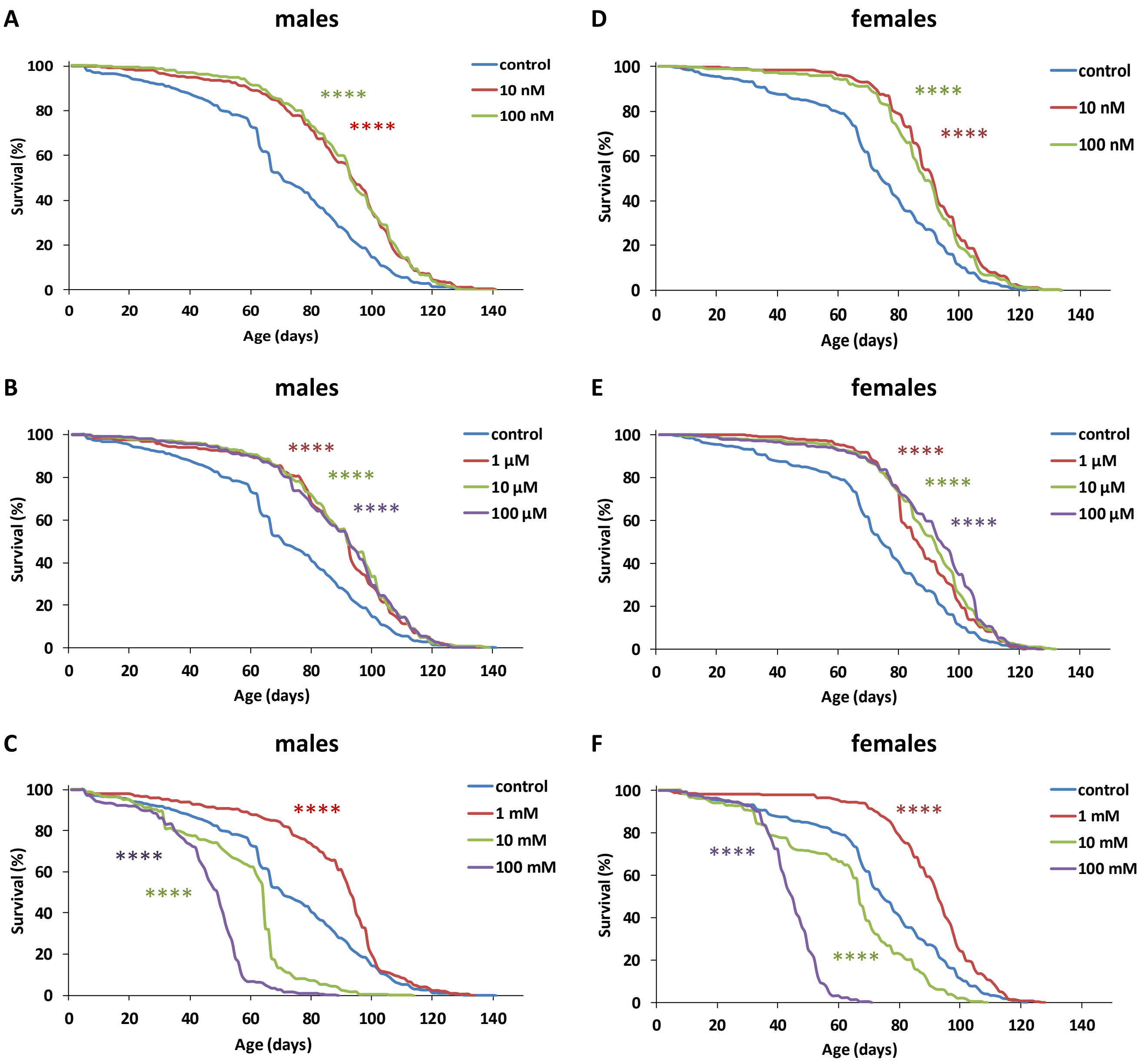

Supplementary Figure S2. Influence of NAC supplementation with nano- (A, D), micro- (B, E) and (C, F) millimolar concentrations on the *D. virilis* male (A, B, C) and female (D, E, F) lifespan (combined data of two independent replications). *p<0.05, **p<0.01, ***p<0.001, ****p <0.0001, Kolmogorov–Smirnov test.

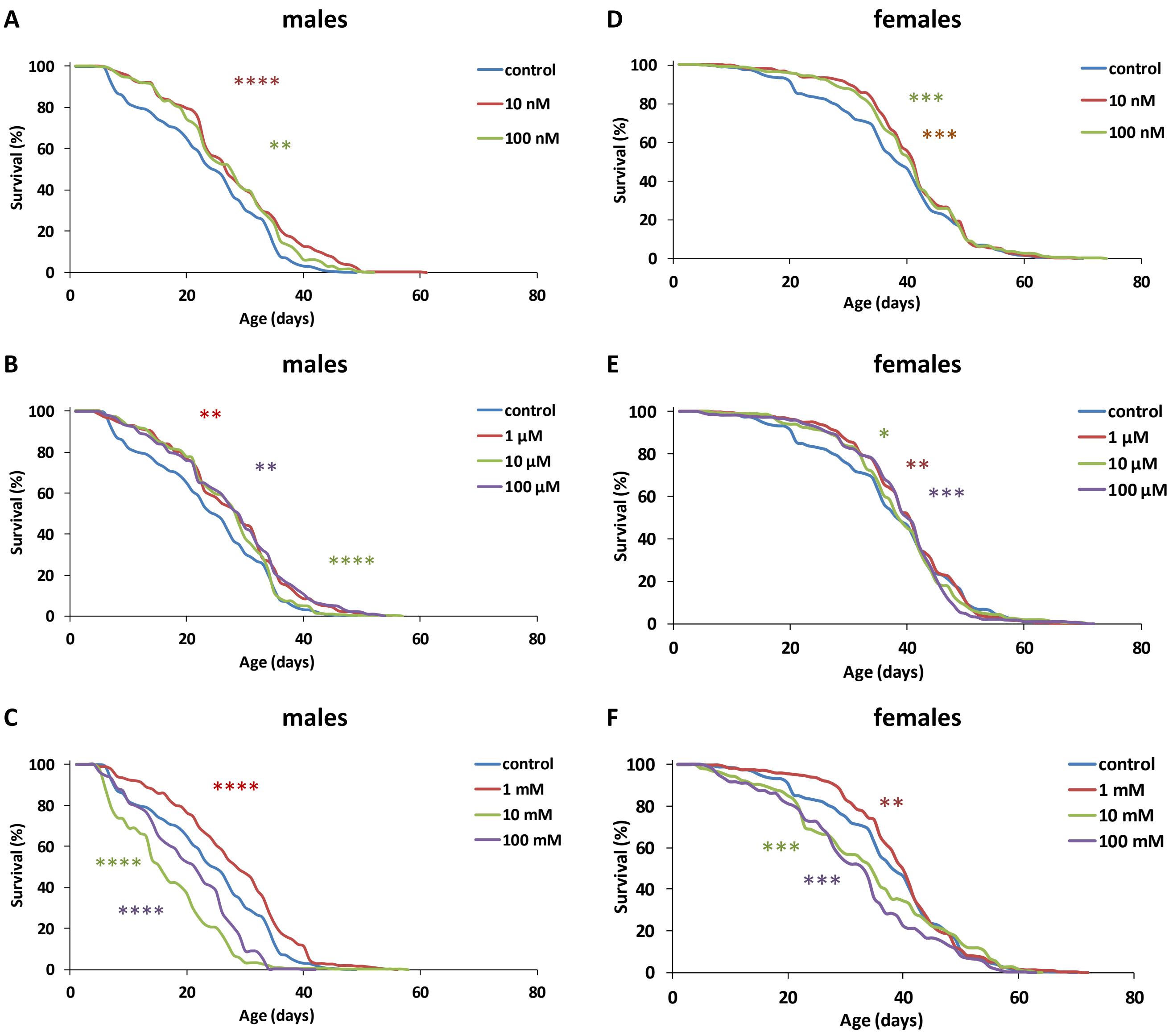

Supplementary Figure S3. Influence of NAC supplementation with nano- (A, D), micro- (B, E) and (C, F) millimolar concentrations on the *D. kikkawai* male (A, B, C) and female (D, E, F) lifespan (combined data of two independent replications). *p<0.05, **p<0.01, ***p<0.001, ****p <0.0001, Kolmogorov–Smirnov test.
