# Supplementary material for: Effects of N-acetyl-L-cysteine on lifespan, locomotor activity and stress-resistance of 3 Drosophila species with different lifespans
Source: Aging (Albany NY). 2018 Sep 20;10(9):2428–58. doi: 10.18632/aging.101561 (PMC6188487; doi:10.18632/aging.101561)
Supplement: Supplementary Tables [file aging-10-101561-s002.pdf]

SUPPLEMENTARY TABLES

Supplementary Table S1. The effects of NAC on *D. melanogaster* lifespan parameters.

Variant	Sex	M (days)	dM (%)	Log-Rank test (p)	Max (days)	dMax (%)	Wang-Allison test (p)	MRDT (days)	dMRDT (%)	n
control	♂	54			64			6.9		535
10 nM	♂	57	5.6	$p < 0.05$	67	4.7	$p < 0.01$	7.2	4.3	306
100 nM	♂	59	9.3	$p < 0.0001$	69	7.8	$p < 0.0001$	6.9	0	296
1 µM	♂	58	7.4	$p < 0.001$	67	4.7	$p < 0.01$	7.8	13	297
10 µM	♂	56	3.7	$p > 0.05$	68	6.2	$p < 0.01$	8.3	20.3	299
100 µM	♂	57	5.6	$p < 0.001$	71	10.9	$p < 0.0001$	8.7	26.1	299
1 mM	♂	56	3.7	$p < 0.05$	65	1.6	$p < 0.05$	8.2	18.8	304
10 mM	♂	44	-18.5	$p < 0.0001$	61	-4.7	$p > 0.05$	6.5	-5.8	271
100 mM	♂	51	-5.6	$p < 0.001$	61	-4.7	$p > 0.05$	6.1	-11.6	269
control	♀	67			78			6.5		583
10 nM	♀	66	-1.5	$p < 0.001$	74	-5.1	$p < 0.01$	6	-7.7	312
100 nM	♀	66	-1.5	$p < 0.0001$	74	-5.1	$p < 0.0001$	6.6	1.5	306
1 µM	♀	67	0	$p > 0.05$	81	3.8	$p > 0.05$	7.1	9.2	276
10 µM	♀	64	-4.5	$p < 0.001$	73	-6.4	$p < 0.001$	6.7	3.1	307
100 µM	♀	64	-4.5	$p < 0.001$	76	-2.6	$p < 0.05$	7.3	12.3	295
1 mM	♀	64	-4.5	$p < 0.0001$	73	-6.4	$p < 0.0001$	6.7	3.1	295
10 mM	♀	64	-4.5	$p > 0.05$	76	-2.6	$p > 0.05$	6.7	3.1	289
100 mM	♀	60	-10.4	$p < 0.0001$	71	-9	$p < 0.0001$	6.5	0	277

M - median lifespan; Max - maximum lifespan (age of 90% mortality); MRDT - mortality rate doubling time; dM, dMax, dMRDT - differences between median and maximum (age of 90% mortality) lifespan and MRDT of control and experimental flies, respectively; n - number of flies.

Supplementary Table S2. The effects of NAC on *D. virilis* lifespan parameters.

Variant	Sex	M (days)	dM (%)	Log-Rank test (p)	Max (days)	dMax (%)	Wang-Allison test (p)	MRDT (days)	dMRDT (%)	n
control	♂	71			105			18.2		588
10 nM	♂	94	32.4	$p < .0001$	114	8.6	$p < 0.0001$	13.3	-26.9	287
100 nM	♂	93	31	$p < .0001$	114	8.69	$p < 0.0001$	12	-34.1	285
1 µM	♂	93	31	$p < .0001$	113	7.6	$p < 0.0001$	12.6	-30.8	287
10 µM	♂	92	29.6	$p < .0001$	113	7.6	$p < 0.0001$	12.4	-31.9	288
100 µM	♂	93	31	$p < .0001$	113	7.6	$p < 0.0001$	12.4	-31.9	282
1 mM	♂	93	31	$p < .0001$	108	2.9	$p > 0.05$	12.2	-33	293
10 mM	♂	65	-8.5	$p < .0001$	74	-29.5	$p < 0.0001$	12.8	-29.7	328
100 mM	♂	50	-29.6	$p < .0001$	58	-44.8	$p < 0.0001$	9.9	-45.6	276
control		75			102			14.7		640
10 nM	♀	92	22.6	$p < .0001$	109	6.9	$p < 0.0001$	10.2	-30.6	314
100 nM	♀	89	18.7	$p < .0001$	107	4.9	$p < 0.001$	10.7	-27.2	293
1 µM	♀	87	16	$p < .0001$	109	6.9	$p < 0.01$	10.2	-30.6	330
10 µM	♀	92	22.7	$p < .0001$	109	6.9	$p < 0.0001$	10.7	-27.2	299
100 µM	♀	94.5	26	$p < .0001$	112	9.8	$p < 0.0001$	10.2	-30.6	320
1 mM	♀	93	24	$p < .0001$	111	8.8	$p < 0.0001$	9.4	-36.1	287
10 mM	♀	67	-10.7	$p < .0001$	90	-11.8	$p < 0.0001$	14.4	-2	445
100 mM	♀	45	-40	$p < .0001$	54	-47.1	$p < 0.0001$	6.4	-56.5	438

M - median lifespan; Max - maximum lifespan (age of 90% mortality); MRDT - mortality rate doubling time; dM, dMax, dMRDT - differences between median and maximum (age of 90% mortality) lifespan and MRDT of control and experimental flies, respectively; n - number of flies.

Supplementary Table S3. The effects of NAC on *D. kikkawai* lifespan parameters.

Variant	Sex	M (days)	dM (%)	Log-Rank test (p)	Max (days)	dMax (%)	Wang-Allison test (p)	MRDT (days)	dMRDT (%)	n
control	♂	25			36			7.5		538
10 nM	♂	27	8	p < 0.0001	43	19.4	p < 0.0001	8.4	12	298
100 nM	♂	28	12	p < 0.0001	39	8.3	p < 0.01	7.1	-5.3	292
1 µM	♂	29	16	p < 0.0001	40	11.1	p < 0.0001	7.5	0	285
10 µM	♂	29	16	p < 0.01	36	0	p > 0.05	6.4	-14.7	299
100 µM	♂	29	16	p < 0.0001	41	13.9	p < 0.0001	7.9	5.3	312
1 mM	♂	29	16	p < 0.0001	41	13.9	p < 0.0001	7.4	-1.3	292
10 mM	♂	16	-36	p < 0.0001	28	-22.2	p < 0.0001	10.2	36	293
100 mM	♂	22	-12	p < 0.0001	30	-16.7	p < 0.0001	5.9	-21.3	296
control	♀	39			51			7.9		641
10 nM	♀	41	5.1	p < 0.05	51	0	p > 0.05	6.4	-19	293
100 nM	♀	41	5.1	p < 0.05	50	-2	p > 0.05	7.4	-6.3	295
1 µM	♀	41	5.1	p > 0.05	51	0	p > 0.05	6.5	-17.7	297
10 µM	♀	39	0	p > 0.05	50	-2	p > 0.05	7.6	-3.8	299
100 µM	♀	40.5	3.8	p > 0.05	48	-5.9	p < 0.01	6.8	-13.9	290
1 mM	♀	40	2.6	p > 0.05	51	0	p > 0.05	7.1	-10.1	311
10 mM	♀	35	-10.3	p > 0.05	55	7.8	p < 0.05	10.3	30.4	296
100 mM	♀	34	-12.8	p < 0.0001	50	-2	p > 0.05	9.6	21.5	288

M - median lifespan; Max - maximum lifespan (age of 90% mortality); MRDT - mortality rate doubling time; dM, dMax, dMRDT - differences between median and maximum (age of 90% mortality) lifespan and MRDT of control and experimental flies, respectively; n - number of flies.

Supplementary Table S4. Influence of NAC treatment on D. melanogaster resistance to paraquat, starvation and hyperthermia (see Full Text version).

Supplementary Table S5. Influence of NAC treatment on D. virilis resistance to paraquat, starvation and hyperthermia (see Full Text version).

Supplementary Table S6. Influence of NAC treatment on D. kikkawai resistance to paraquat, starvation and hyperthermia (see Full Text version).

Supplementary Table S7. Relative antioxidant and H_2S biosynthesis gene expression levels normalized to control in *D. melanogaster*.

variant	*Cat/CG6871*		*Sod1/CG11793*		*Cbs/CG1753*		*Eip55E/CG5345*		*Nfs1/CG12264*	
	♂	♀	♂	♀	♂	♀	♂	♀	♂	♀
control	1.0	1.0	1.0	1.0	1.0	1.0	1.0	1.0	1.0	1.0
10 nM	1.4 **	0.2*	1.1	0.6*	2.0*	1.1	0.5*	0.4*	3.0*	0.8*
100 nM	1.5 **	0.3*	1.0	0.9	1.3	1.1	0.6*	0.4*	1.2*	0.5*
1 µM	0.8*	0.4*	1.1	0.6*	2.6*	0.7	1.8*	0.5*	5.6**	0.8*
10 µM	0.5*	0.3*	0.7*	0.5*	0.4*	0.6	0.2***	0.7*	0.5*	0.7*
100 µM	5.8* **	0.5*	1.7**	0.5*	5.3**	1.4*	0.5**	2.9**	0.8*	2.1*
1 mM	6.3* **	0.3*	2.8**	0.4**	2.3*	0.4*	0.3***	1.9*	0.8*	2.1*
10 mM	1.7* *	0.5*	1.2*	0.4**	1.5*	0.6	4.9***	0.4*	15.0***	0.5*
100 mM	7.0* **	0.6*	1.6**	0.7*	1.6*	0.7	0.03***	0.1*	3.6**	0.6*

*p<0.05, **p<0.01, ***p<0.001

Supplementary Table S8. List of primers for qRT-PCR.

Gene	Forward	Reverse
β-Tubulin	5'-GGCCAACTGAACGCTGATCT -3'	5'-AAGCCGGGCATGAAGAAGTG-3'
Catalase (*Cat/CG6871*)	5'-CCCAAGAACTACTTTGCTGAGGTG-3'	5'-AGGAGAACAGACGACCATGCAG-3'
Superoxide dismutase 1 (*Sod1/CG11793*)	5'-TGCACGAGTTCGGTGACAACAC-3'	5'-TCCTTGCCATACGGATTGAAGTGC-3'
Cystathionine β-synthase (*Cbs/CG1753*)	5'-AGATTACGCCCAACATCCTC-3'	5'-CTCGCACTTGGCATACATCT-3'
Ecdysone-induced protein 55E (*Eip55E/CG5345*)	5'-GTGTGGATCGAGTCACCAAC-3'	5'-GGAGGTCAGGAAGGTGTTGT-3'
Nfs1 cysteine desulfurase (*Nfs1/CG12264*)	5'-CTTTCTTTGCGTGAGATGGA-3'	5'-GTATGTGGCTTTGGCATCAC-3'
